# 2-Methoxybenzaldehyde effectively repels ants

**DOI:** 10.1093/jee/toad225

**Published:** 2023-12-09

**Authors:** Tomas Kay, Georges Siegenthaler, Timothy Kench, Laurent Keller

**Affiliations:** Department of Ecology and Evolution, University of Lausanne, Lausanne, Switzerland; Sigene Sarl, Acacias, Geneva, Switzerland; Department of Chemistry, Imperial College London, London, UK; Department of Ecology and Evolution, University of Lausanne, Lausanne, Switzerland

**Keywords:** ant repellent, invasive ant, natural chemical control, *Solenopsis invicta*, *Wasmannia auropunctata*

## Abstract

Ants can particularly make for harmful pests, infesting human homes and reducing crop yields. The damage caused by ants and the efforts to mitigate the damage are hugely costly. Broad-spectrum insecticides are used most commonly; however, due to their negative side effects, there is increasing interest in nontoxic alternatives. One promising commercially available alternative is 2-hydroxybenzaldehyde, which is naturally produced by various arthropods as a means of chemical defense and effectively repels ants. Here we conduct a structure–activity relationship investigation, testing how different chemical modifications alter the repellence of 2-hydroxybenzaldehyde. We find that 2-methoxybenzaldehyde is considerably more effective than 2-hydroxybenzaldehyde at repelling the common black garden ant, *Lasius niger*. We next compare the most effective repellent chemicals against 4 particularly harmful ant species to confirm that the results obtained with *L. niger* are general to ants and that our results are relevant to mitigate the costs of ant damage.

## Introduction

Ants can make for harmful pests, infesting homes and destroying crops at huge annual costs (Bourke et al. 1995, Holway 1999, Holway et al. 2002, Lowe et al. 2012, Bertelsmeier et al. 2015). For example, *Solenopsis invicta* (Buren, 1972) and *Wasmannia auropunctata* (Roger, 1863) have cost an estimated 37 and 20 billion US dollars, respectively, since the 1930s. Ninety-two percent of these costs are directly attributable to damage (mostly to agriculture and public and social welfare), and the remainder is mostly attributable to management (Angulo et al. 2021).

One of the reasons that ants can be so harmful is that they live in complex social groups, where different individuals specialize in the performance of different tasks (e.g., foraging, cleaning, and brood care), increasing per capita productivity (Ulrich et al. 2018). This social structure depends on efficient communication, which in ants is primarily chemical, and often involves volatile molecules. Volatiles can, for example, signal the presence of an intruder and the presence and quality of food sources (Wilson 1965, Lopes et al. 2023).

The main strategy to limit ant damage is the use of broad-spectrum insecticides (Klotz et al. 1995, 1997). However, following evidence that nontoxic ant repellents can be equally effective and much safer, there has been considerable effort to identify and apply such chemicals, exploiting the chemical sensitivity of ants to manage their negative impacts (Klotz et al. 1997). For example, repellent chemicals have been applied around window frames to prevent ants from entering homes, around tree-trunks to prevent ants from foraging in crop-bearing trees, and on the packaging of containerized cargo to prevent ants from consuming shipped goods (Shorey et al. 1992, 1996, Hashimoto et al. 2022).

2-Hydroxybenzaldehyde is a particularly promising ant repellent (Boeve´ and Giot 2014). It is effective, nontoxic, naturally occurring, and is already commercially available (Flint and Kaufert 1942). It is synthesized by various arthropods as means of chemical defense (Eisner et al. 1963, Pauls et al. 2016). The synthesis requires salicin, which arthropods sequester from plants. For example, leaf beetle larvae secrete 2-hydroxybenzaldehyde in response to the threat of predation, and they synthesize the 2-hydroxybenzaldehyde from salicin taken up from poplar trees (Kuhn et al. 2004, Boeckler et al. 2011, Erb and Robert 2016). Structurally, 2-hydroxybenzaldehyde comprises a benzine ring with an aldehyde group (a benzaldehyde) and a hydroxy group at the second carbon of the benzene ring. For 2-hydroxybenzaldehyde to repel ants, it necessarily activates one or more odorant receptors (Hallem et al. 2006). The strength of the subsequent behavioral response depends on how well the chemical structure of 2-hydroxybenzaldehyde binds with that (those) of the receptor(s).

The identity and structure of the odorant receptor activated by 2-hydroxybenzaldehyde are unknown. But by measuring the different responses elicited by structurally similar chemicals, it is possible to make circumstantial inferences about the receptor’s structure and thereby identify more effective repellent chemicals. Here, using lab colonies of the common black garden ant (*Lasius niger* (Linnaeus, 1758)), we tested the repellence of a broad panel of chemicals that are structurally similar to 2-hydroxybenzaldehyde. We identified several previously unapplied chemicals with repellent function, including one that is considerably more effective: 2-methoxybenzaldehyde. Next, using 4 economically costly ant species (*W. auropunctata*, *Paratrechina longicornis* (Latreille, 1802), *S. invicta*, and *Tapinoma magnum* (Mayr, 1861)), we confirmed that the results obtained with *L. niger* were general across ant subfamilies and relevant to combating ant damage.

## Methods

### Repellent Chemicals

**Fig. 1.**
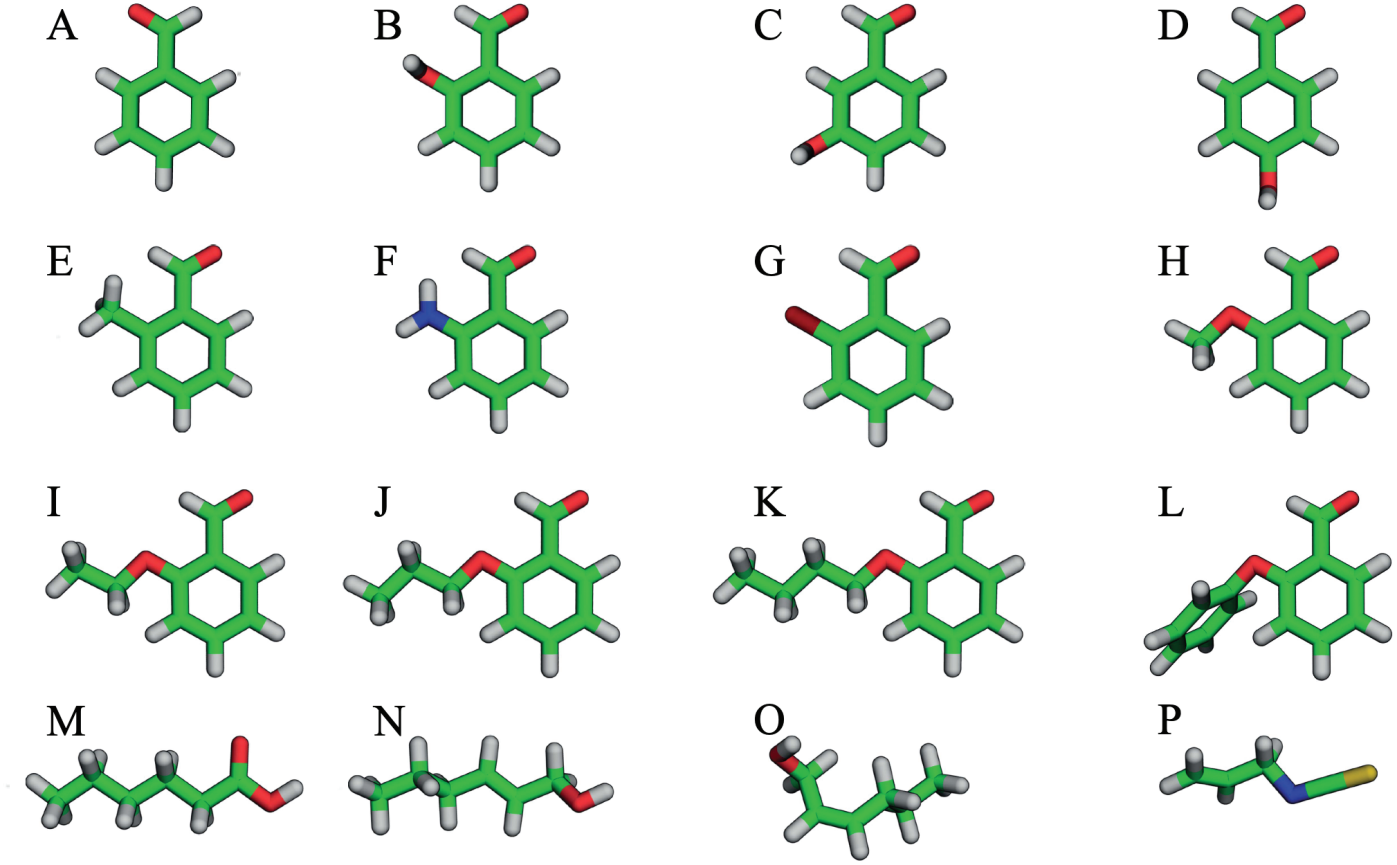
Chemical structures. A) Benzaldehyde; B) 2-hydroxybenzaldehyde; C) 3-hydroxybenzaldehyde; D) 4-hydroxybenzaldehyde; E) 2-methylbenzaldehyde; F) 2-aminobenzaldehyde; G) 2-bromobenzaldehyde; H) 2-methoxybenzaldehyde; I) 2-ethoxybenzaldehyde; J) 2-propoxybenzaldehyde; K) 2-butoxybenzaldehyde; L) 2-phenoxybenzaldehyde; M) cis-2-hexenol; N) trans-2-hexenol; O) hexanoic axid; P) AITC.

To make inferences about the structure of the odorant receptor underlying the behavioral response to 2-hydroxybenzaldehyde (98%; from Merck [S356-100G]), we investigated the effect of various structural modifications on the strength of the behavioral response:

- Moving the hydroxy-group around the benzene ring, testing 3-hydroxybenzaldehyde (*≥* 99%; from Merck [H19808-5G]) and 4-hydroxybenzaldehyde (*≥*95%; from Merck [H19808-5G]), the other 2 isomers of hydroxybenzaldehyde (Fig. 1).
- Replacing the hydroxy-group with functional groups of differing size and/or polarity: benzaldehyde (i.e., no group added; *≥*99.6%; from Merck [8017560100]), 2-methylbenzaldehyde (97%; from Merck [117552-25G]), 2-aminobenzaldehyde (*≥*98%; from Merck [A9628-100MG]), 2-bromobenzaldehyde (98%; from Merck [B57001-10G]) (Fig. 1).
- Attaching alkyl chains of various lengths to the hydroxy group: 2-methoxybenzaldehyde (98%; from Merck [109622-2G]), 2-ethoxybenzaldeyhde (*≥*97%; from Merck [153729-25G]), 2-n-propoxybenzaldehyde (*≥*97%; from Alfa Aesar [AAH50473MD]), 2-butoxybenzaldehyde (95%; from abcr GmbH [AB404676]), and 2-phenoxybenzaldehyde (from Merck [CDS013719-500MG]) (Fig. 1).

We additionally tested 3 aliphatic alcohols: trans-2-hexen-1-ol (*≥*95%; from Merck [W256218-SAMPLE-K]), cis-2-hexen-1-ol (AKos Consulting & Solutions [AKOS006229286]) and hexanoic acid (*≥* 99%; from Merck [153745-2.5G]) (Fig. 1).

Finally, to benchmark the chemicals tested here, we included allyl isothiocyanate (AITC—95%; from Merck (377430-5G]), which has recently been found to repel *S. invicta* (Hashimoto et al. 2019), and citrus essential oil, which is commonly referred to as a DIY solution for ant infestations (e.g., www.goodhousekeeping.com/home/cleaning/a20707344/homemade-natural-ant-spray/ or www.saferbrand.com/articles/natural-ant-killer).

All tested chemicals were used at 5% concentration in a standard solvent comprising 96% ethanol (60%), isopropyl myristate (w/w 30%), dipropylene glycol (7.5%), fixer: hercolyn D-E, methyl hydrogenated rosinate (1%), UVB absorber: Parsol MVX (1%), and antioxidant: butylated hydroxytoluene (0.5%) (all from Essencia AG, CH-8409 Winterthur). This solvent constituted the negative control.

### Experimental Design

In the first experiment, we assayed the repellence of all chemicals mentioned above using 30 colonies of *L. niger*, a species that is found across Europe, parts of North America and Asia. These colonies were established from foundress queens collected in Lausanne between 2013 and 2017 and maintained thereafter in the lab at 26 °C under a 12h:12h light:dark cycle. The colonies (and those of the other species tested) were housed in open plastic boxes with Fluon-coated walls and had ad libitum access to water tubes bunged with cotton wool, and were fed weekly with dead flies and a standard artificial ant food (Bhatkar and Whitcomb 1970).

In preliminary experiments, we placed food on top of small stakes in the boxes that housed the ant colonies and applied the chemical in a band around the stakes. We then measured the number of ants at the food source after a given time as an indication of the strength of repellence, similar to the setup of (Duangphakdee et al. 2009). However, we found that even with no repellent there was large variation in foraging effort—possibly depending on the demography, activity level, or nutritional status of the colony. Consequently we developed an alternative bioassay in which, for a given trial, 300 µl of the chemical solution was applied uniformly to a square paper perimeter, with a width of 5 mm, and internal dimensions of 100 mm × 100 mm. Five workers were randomly selected from the colony with fine insect forceps, and transferred to a Petri dish with Fluon-lined walls. The Petri dish was carefully inverted at the center of the 100 mm × 100 mm area enclosed within the paper perimeter, and left for 1 min to allow the ants to calm. The Petri dish was then gently lifted. In this setup, the workers would repeatedly explore out from the center and turn back when they encountered the repellent perimeter. Depending on the strength of the repellent effect it would take more or less time for the workers to eventually cross the perimeter, and the median time until crossing the perimeter (i.e., when ant 3 of 5 had exited the square) was recorded for each trial. Trials were stopped after 20 min if the third ant had not yet crossed the border. Using this bioassay each colony was tested once with each chemical (*n* = 30).

In a second experiment, using the 4 most repellent chemicals identified in the first experiment (2-hydroxybenzaldehyde, 2-bromobenzaldehyde, 2-ethoxybenzaldehyde, and 2-methoxybenzaldehyde) and the negative control of solvent only, we assayed the generality of our findings across ant species. We repeated the same experimental protocol, using lab stock colonies of *W. auropunctata* (native to Central and South America; invasive in tropical regions globally), *P. longicornis* [native to India and Nepal; invasive in tropical regions globally (Tseng et al. 2023)], *S. invicta* (native to South America; invasive globally), and *T. magnum* (native to North Africa; invasive to Europe (Seifert et al. 2017)). For each of these species, 20 replicate trials were run for each chemical (*n* = 20), for which workers were sampled equally, and without replacement from across available stock colonies (approximately 5 per species) ensuring that workers from a given stock colony were tested the same number of times under all 5 conditions.

Since *W. auropunctata* was generally slower to cross the perimeter than *L. niger*, and in most cases, no workers had crossed the perimeter after 20 min, the trials for this species were extended to last for 2 h.

### Statistics

To compare the efficacy of the different repellents we used survival analysis. These approaches were designed to compare how different medical treatments influence patient survival, but effectively compare the duration of time taken until the occurrence of an event under different conditions. Importantly, survival analysis can account for censored data (i.e., instances when the event did not occur within the time frame of observation) where rank tests cannot (Bradburn et al. 2003). To make statistical comparisons we used Cox regressions (David 1972) using the *R* packages *Survival* and *Survminer* (Kassambara et al. 2017, Therneau and Lumley 2015). Survival curves were calculated using functions *Surv* and *Survfit*, and plotted using the function *ggsurvplot*.

## Results

### Testing the Repellence of a Broad Panel of Structurally Similar Chemicals

**Fig. 2.**
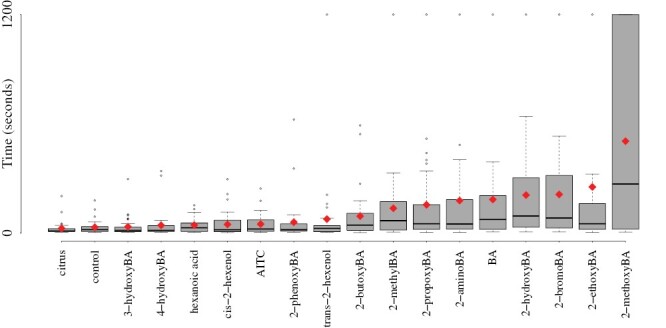
Testing a broad panel of molecules and mixtures. Most of the chemicals that we tested are structurally similar to 2-hydroxyBA, an established insect repellent, but we also included AITC and citrus essential oil to benchmark these chemicals against a recent study and an example DIY solution. Plotted are the distributions of median duration until exit of the chemical perimeter, over 30 trials for each chemical. The stronger the repellence, the longer the duration. Mean values are indicated with red points.

We measured repellence as the median duration of time taken for ants to cross a paper perimeter that had been saturated with a repellent solution. The longer the duration of time, the more effective the repellent. We used survival analysis to statistically compare the effectiveness of different repellents. Our first experiment confirmed that 2-hydroxybenzaldehyde was an effective repellent against *L. niger*; 4-fold more effective than the negative control (Figs. 2 and 4; Cox proportional-hazard model hazard ratio vs. control: *z* = 4.59; df = 1; *P* *< *0.001). Repellence was completely lost when the –OH group was moved to the third and fourth carbon, indicating that the positioning of the group at the second carbon is critical to odorant receptor activation (Cox proportional-hazard model hazard ratio vs. control for 3-hydroxyBA: *z* = 0.176; df = 2; *P* = 0.861 and for 4-hydroxyBA: *z* = −0.301; df = 2; *P* = 0.763).

We next investigated whether the hydroxy-group was essential for repellence by replacing the hydroxy-group with functional groups of differing size and/or polarity, comparing 2-hydroxyBA, 2-bromoBA, 2-aminoBA, 2-methoxyBA, 2-methylBA, and BA. Among these chemicals, 2-methoxyBA appeared to be significantly and substantially more effective than 2-hydroxybenzaldehyde (Figs. 2 and 4; Cox proportional-hazard model hazard ratio vs. 2-hydroxyBA: *z* = 2.35; df = 5; *P* = 0.0189), and all other chemicals tested Fig. 2: Cox proportional-hazard model hazard ratio vs. 2-methoxyBA for 2-bromobenzaldehyde: *z* = 2.41; df = 5; *P* = 0.0161, for 2-aminobenzaldehyde: *z* = 2.99; df = 5; *P* = 0.0028, for 2-methylbenzaldehyde: *z* = 3.46; df = 5; *P* *< *0.001, and for benzaldehyde = *z* =; df = 5; *P* = 0.0044.

Since 2-methoxyBA contains a carbon chain of length one, we next varied the length of this carbon chain. Attaching alkyl chains of increasing length to the hydroxy group monotonically decreased the repellence, with 2-methoxyBA being most effective, followed by 2-ethoxyBA, 2-propoxyBA, 2-butoxyBA, and finally 2-phenoxyBA (Figs. 2 and 4; Cox proportional-hazard model hazard ratio vs. 2-methoxyBA for 2-ethoxyBA: *z* = 2.01; df = 4; *P* = 0.045, for 2-propoxyBA: *z* = 2.76; df = 4; *P* = 0.0068, for 2-butoxyBA: *z* = 3.63; df = 4; *P* *< *0.001, and for 2-phenoxyBA: *z* = 4.83; df = 4; *P* *< *0.001).

None of the more structurally different chemicals (cis-2-hexenol, trans-2-hexenol, and hexanoic acid) were significantly more repellent than the control (Figs. 2 and 4; Cox proportional-hazard model hazard ratio vs. control for cis-2-hexenol: *z* = −0.857; df = 3; *P* = 0.392, for trans-2-hexenol: *z* = −1.01; d = 3; *P* = 0.313, and for hexanoic acid: *z* = −0.902; d = 3; *P* = 0.367). These aliphatic alcohols are known to repel insects, but it is likely that the effects are so much weaker than the chemicals with a complete benzene ring that their repellence is not detectable at the concentration used here.

Finally, completely differently structured chemicals (AITC) and mixtures (citrus essential oil) which have previously been found to repel ants were also not significantly more effective than the control (Figs. 2 and 4; Cox proportional-hazard model hazard ratio vs. control for AITC: *z* = −1.19; df = 2; *P* = 0.236, and for citrus essential oil: *z* = 0.806; df = 2; *P* = 0.42).

### Responses are Consistent Across Ant Species

The 4 most repellent chemicals identified in experiment 1 (2-hydroxyBA, 2-bromoBA, 2-ethoxyBA, and 2-methoxyBA) were tested against 4 additional ant species from 3 ant subfamilies to establish (i) whether the receptor mediating the behavioral response of *L. niger* was common across ants, and hence (ii) whether the chemicals shown to be effective here could be useful in mitigating ant damage. The responses of the other species closely paralleled those of *L. niger*.

**Fig. 3.**
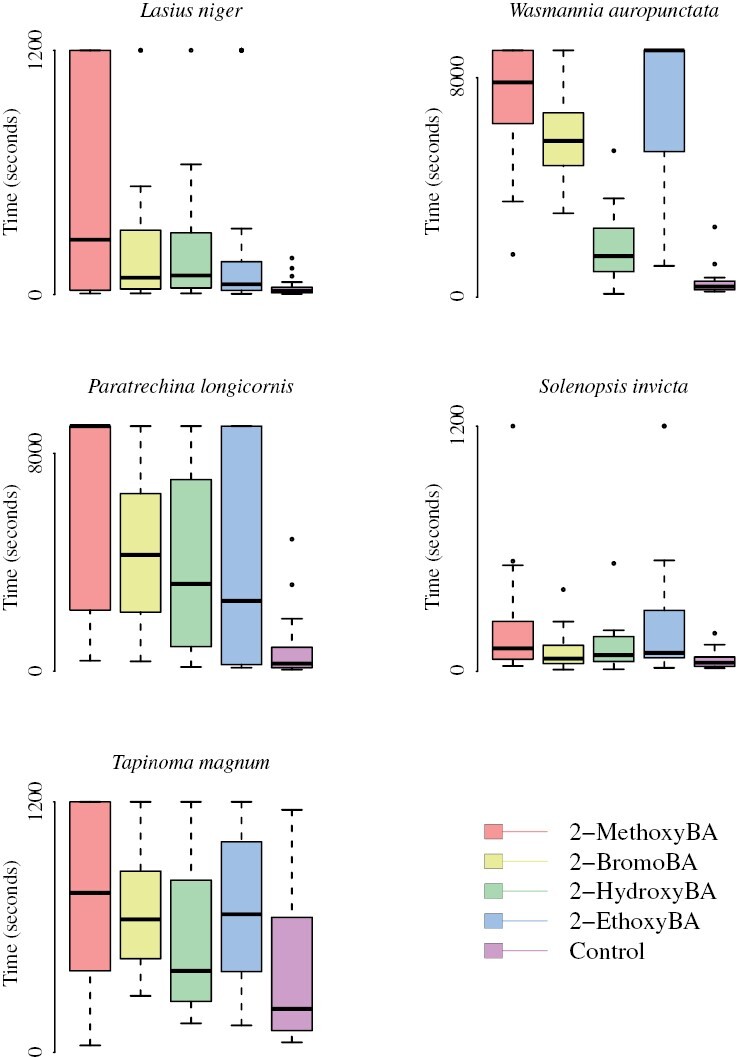
Testing the most effective repellent chemicals against harmful ant species. The first experiment revealed that 2-hydroxyBA, 2-bromoBA, 2-ethoxyBA, and 2-methoxyBA were the most effective repellents. We therefore tested these against various harmful species to establish whether the relevant receptor was conserved and therefore whether the identified chemicals could be relevant to combating harmful species. Plotted are the distributions of median duration until the exit of the chemical perimeter, over 20 trials for each chemical. The stronger the repellence, the longer the duration. The results are broadly consistent across species.

For *W. auropunctata*, all of the 4 chemicals were significantly more effective than the negative control (Figs. 3 and 5; Cox proportional-hazard model hazard ratio vs. control for 2-hydroxyBA: *z* = −4.6; df = 4; *P* < 0.001, for 2-methoxyBA: *z* = −9.11; df = 4; *P* *< *0.001, for 2-ethoxyBA: *z* = −9.23; df = 4; *P* < 0.001, and for 2-bromoBA: *z* = −8.06; df = 4; *P* *< *0.001). As with *L. niger*, 2-hydroxyBA was the least effective of the 4 (Cox proportional-hazard model hazard ratio vs. 2-hydroxyBA for 2-methoxyBA: *z* = −7.04; df = 3; *P* *< *0.001, for 2-ethoxyBA: *z* = −7.25; df = 3; *P* *< *0.001, and for 2-bromoBA: *z* = −5.66; df = 3; *P* *< *0.001), followed by 2-bromoBA (Cox proportional-hazard model hazard ratio vs. 2-bromoBA for 2-methoxyBA: *z* = −2.64; df = 2; *P* = 0.008, and for 2-ethoxyBA: *z* = −3.26; df = 2; *P* = 0.001). However, the difference between 2-methoxyBA and 2-ethoxyBA was not statistically significant (Cox proportional-hazard model hazard ratio vs. 2-methoxyBA for 2-ethoxyBA: *z* = −0.959; df = 1; *P* = 0.337).

For *P. longicornis*, all of the 4 chemicals were significantly more effective than the negative control (Figs. 3 and 6; Cox proportional-hazard model hazard ratio vs. control for 2-hydroxyBA: *z* = −4.2; df = 4; *P* *< *0.001, for 2-methoxyBA: *z* = −5.47; df = 4; *P* *< *0.207, for 2-ethoxyBA: *z* = −4.22; df = 4; *P* *< *0.001, and: *z* for 2-bromoBA = −4.49; df = 4; *P* *< *0.001). Consistent with *L. niger*, 2-methoxyBA was significantly more effective than 2-hydroxyBA (Cox proportional-hazard model hazard ratio vs. 2-hydroxyBA for 2-methoxyBA: *z* = −2.05; df = 3; *P* = 0.041), and otherwise the differences between 2-hydroxyBA, 2-methoxyBA, 2-ethoxyBA, and 2-bromoBA were not statistically significant.

For *S. invicta*, all of the 4 chemicals were significantly more effective than the negative control (Figs. 3 and 7; Cox proportional-hazard model hazard ratio vs. control for 2-hydroxyBA: *z* = −2.47; df = 4; *P* = 0.014, for 2-methoxyBA: *z* = −3.83; df = 4; *P* *< *0.001, for 2-ethoxyBA: *z* = −4.04; df = 4; *P* *< *0.001, and for 2-bromoBA: *z* = −2.02; df = 4; *P* = 0.043). Consistent with *L. niger*, 2-ethoxyBA was significantly more effective than 2-bromoBA (Cox proportional-hazard model hazard ratio vs. 2-bromBA for 2-ethoxyBA: *z* = −2.12; df = 2; *P* = 0.034), and otherwise, the differences between 2-hydroxyBA, 2-methoxyBA, 2-ethoxyBA, and 2-bromoBA were not statistically significant.

**Fig. 4.**
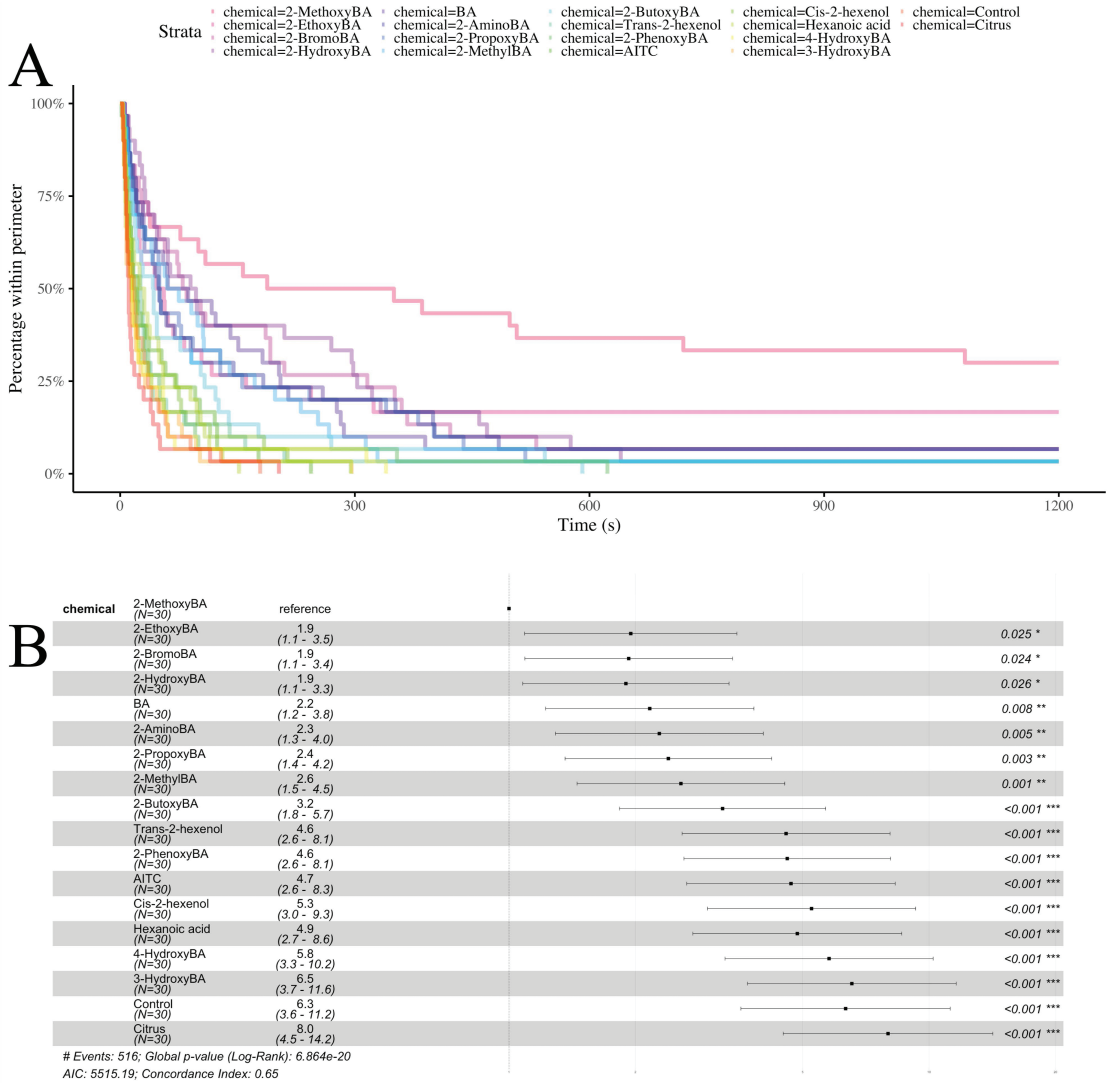
Survival analysis of *L. niger* data. A) Survival curves for the seventeen active chemicals and the negative control. B) Statistical comparisons of the survival curves based on Cox proportional-hazard models. For each chemical, the point indicates the hazard ratio relative to the reference (set here to be methoxyBA). Confidence intervals for the hazard ratios are plotted, and *P*-values are given.

**Fig. 5.**
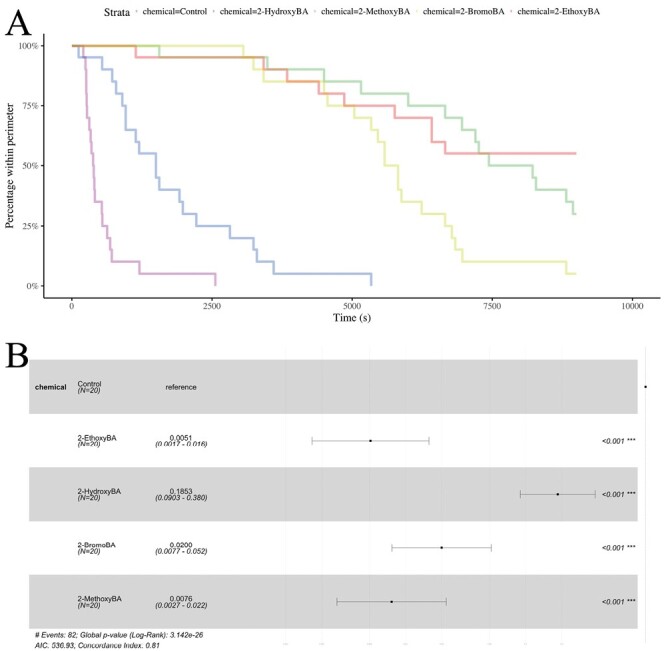
Survival analysis of *W. auropunctata* data. A) Survival curves for the 4 active chemicals and the negative control. B) Statistical comparisons of the survival curves based on Cox proportional-hazard models. For each chemical, the point indicates the hazard ratio relative to the reference (the negative control). Confidence intervals for the hazard ratios are plotted, and *P*-values are given.

**Fig. 6.**
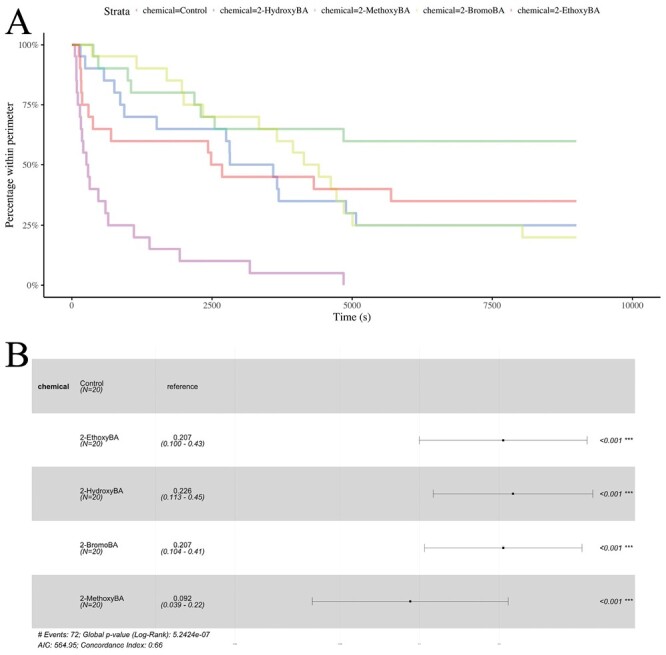
Survival analysis of *P. longicornis* data. A) Survival curves for the 4 active chemicals and the negative control. B) Statistical comparisons of the survival curves based on Cox proportional-hazard models. For each chemical, the point indicates the hazard ratio relative to the reference (the negative control). Confidence intervals for the hazard ratios are plotted, and *P*-values are given.

**Fig. 7.**
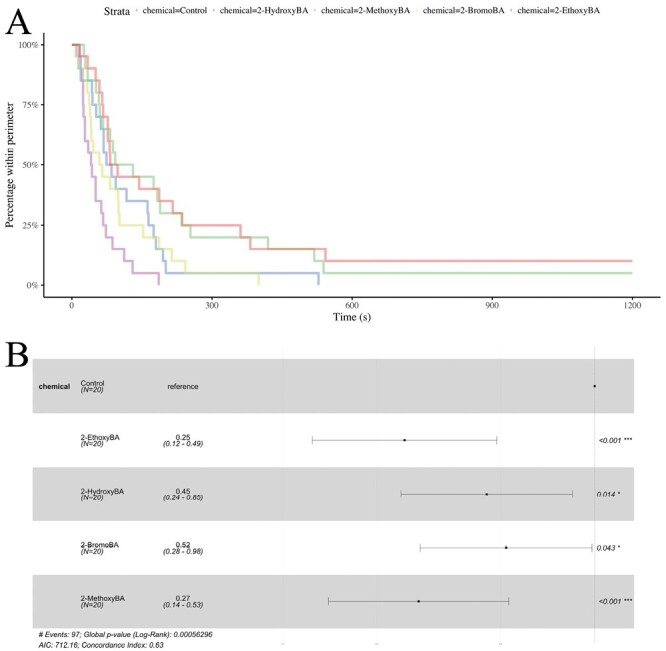
Survival analysis of *S. invicta* data. A) Survival curves for the 4 active chemicals and the negative control. B) Statistical comparisons of the survival curves based on Cox proportional-hazard models. For each chemical, the point indicates the hazard ratio relative to the reference (the negative control). Confidence intervals for the hazard ratios are plotted, and *P*-values are given.

**Fig. 8.**
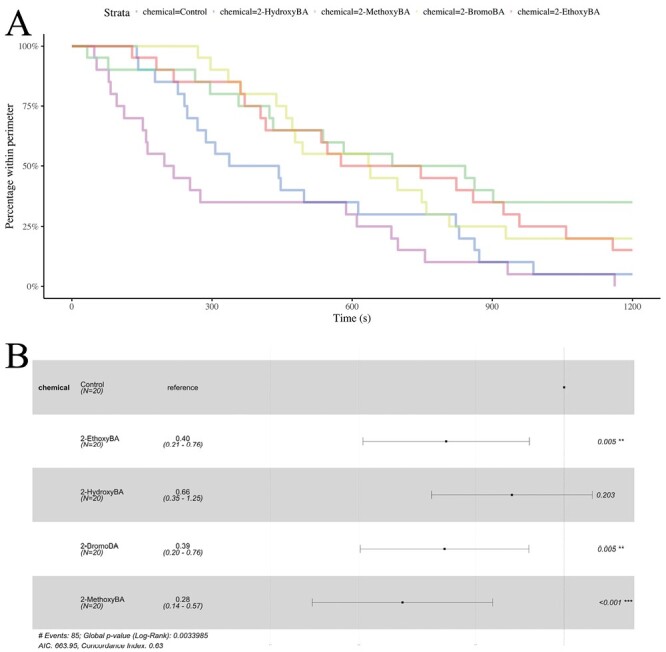
Survival analysis of *T. magnum* data. A) Survival curves for the 4 active chemicals and the negative control. B) Statistical comparisons of the survival curves based on Cox proportional-hazard models. For each chemical, the point indicates the hazard ratio relative to the reference (the negative control). Confidence intervals for the hazard ratios are plotted, and *P*-values are given.

Finally, for *T. magnum*, 2-methoxyBA, 2-bromoBA, and 2-ethoxyBA, but not 2-hydroxyBA, was significantly more effective than the negative control (Figs. 3 and 8; Cox proportional-hazard model hazard ratio vs. control for 2-hydroxyBA: *z* = −1.27; df = 4; *P* = 0.203, for 2-methoxyBA: *z* = −3.51; df = 4; *P* *< *0.001, for 2-ethoxyBA: *z* = −2.78; df = 4; *P* = 0.0055, and for 2-bromoBA: *z* = −2.78; df = 4; *P* = 0.0055). Consistent with *L. niger*, 2-methoxyBA was significantly more effective than 2-hydroxyBA (Cox proportional-hazard model hazard ratio vs. 2-bromBA for 2-ethoxyBA: *z* = −2.45; df = 3; *P* = 0.0143), and otherwise, the differences 2-hydroxyBA, 2-methoxyBA, 2-ethoxyBA, and 2-bromoBA were not statistically significant.

While many pairwise differences were not significantly different, all of the significant differences as well as the trends generally matched the results obtained for *L. niger*.

## Discussion

The objective of this study was to identify nontoxic ant repellents with improved efficacy compared with what is currently available on the market. We used 2-hydroxybenzaldehyde as a starting point because it is a well-known nontoxic ant repellent. Previous studies have found that ants quickly withdraw when sprayed with 2-hydroxybenzaldehyde by chrysomelid larvae (Wallace and Blum 1969, MATSUDA and SUGAWARA 1980) and avoid feeding on sugar water when 2-hydroxybenzaldehyde was added (MATSUDA and SUGAWARA 1980).

We used *L. niger* to conduct a structure–activity relationship investigation, modifying 2-hydroxybenzaldehyde in various ways to infer which aspects of its chemical structure are most important to its repellence and therefore which other chemicals may also be effective repellents. We found several novel repellents and found in particular that 2-methoxybenzaldehyde was significantly and substantially more effective than 2-hydroxybenzaldehyde.

We incorporated various additional chemicals to benchmark the efficacy of the benzaldehydes tested here. We found that citrus essential oil (commonly referred to as DIY solution) was not significantly more repellent than the negative control at the tested concentration. We also included AITC, which was recently found to be a strong ant repellent (Hashimoto et al. 2019). This study placed polyethene strips that either did or did not contain microencapsulated AITC into baited traps and measured the number of foragers inside the trap after 40 min. The average number of foragers inside AITC traps was 0, while the average in control traps was over 150. However, at the concentration used here and in our solvent AITC was not significantly more effective than the negative control, highlighting the comparative efficacy of the benzaldehydes. It is possible that the relative efficacy would be different.

We next compared the efficacy of the 4 most effective chemicals using 4 additional species (*P. longicornis*, *S. invicta*, *W. auropunctata*, and *T. magnum*). Across species, the significant differences and general trends matched those observed for *L. niger*, indicating that that the same receptor likely mediates the avoidance response in all 5 species. These 5 species represent 3 of the most species-rich ant subfamilies (Formicinae, Myrmicinae, and Dolichoderinae), which collectively comprise approximately 80% of ant species (Borowiec et al. 2021), meaning that the results reported here should be relevant to most if not all ant species.

In conclusion, 2-methoxyBA holds great potential as an effective nontoxic ant repellent and could be used, for example, around the trunks of crop-bearing trees, around entry points to human homes, and in the packaging of containerized cargo.

## Data Availability

Raw data and code are available on GitHub at https://github.com/MO-Katy/AntRepellent. The use of 2-methoxybenzaldehyde as an ant repellent is under patent application (Application number: EP22211720.2).
